# Effects of Digital Health Interventions on Functional and Psychological Outcomes in Older Patients With Hip Fractures: Systematic Review and Meta-Analysis of Randomized Controlled Trials

**DOI:** 10.2196/79563

**Published:** 2026-03-12

**Authors:** Wei Fan, Qi Zhang, Qunfeng Lu

**Affiliations:** 1 School of Medicine, Tianjin Tianshi College Tianjin China; 2 Department of Nursing Shanghai Sixth People's Hospital Affiliated to Shanghai Jiao Tong University School of Medicine Xuhui District, Shanghai China

**Keywords:** digital health, older patients, hip fractures, systematic review, meta-analysis

## Abstract

**Background:**

Hip fractures in older adults increasingly challenge public health, making traditional rehabilitation very challenging. Digital health interventions (DHIs) have emerged as a promising solution for postoperative rehabilitation. However, evidence on DHIs’ effects on functional and psychological outcomes remains insufficient.

**Objective:**

This systematic review aimed to comprehensively examine the effects of DHIs on functional and psychological outcomes in older adults with hip fractures.

**Methods:**

Following PRISMA (Preferred Reporting Items for Systematic Reviews and Meta-Analyses) guidelines, we searched 9 databases (PubMed, Embase, CENTRAL, APA PsycINFO, Web of Science, PEDro, CNKI, WANFANG, and SinoMed) from inception to November 13, 2025. Included studies enrolled adults aged 60 years and older with hip fractures, delivered DHIs, assessed functional and psychological outcomes, set usual care or no intervention as the control, and had a randomized controlled trial design. Studies were excluded if they enrolled nonhospitalized patients in the emergency department, patients discharged to nonhome settings, or had inaccessible full text or insufficient data. Study quality was evaluated using the Cochrane Risk of Bias tool 2.0 (Cochrane Collaboration), and evidence certainty was assessed using GRADE (Grading of Recommendations, Assessment, Development and Evaluation). The literature screening, data extraction, and quality assessment were independently conducted by 2 researchers, and any disputes were resolved by the third researcher. We performed analysis using R version 4.0.3 (R Foundation for Statistical Computing) with a random-effects model.

**Results:**

Of 17,723 studies screened, 13 met the inclusion criteria. DHIs, compared to the control, significantly improved hip function (standardized mean difference [SMD] 0.80, 95% CI 0.33-1.26; 95% prediction interval [PI] –0.24 to 1.83; *P=*.007) and functional independence (SMD 1.23, 95% CI 0.34-2.11; 95% PI –0.98 to 3.34; *P*=.02). Despite favorable pooled effects, a wide 95% PI spanning positive or negative values signals substantial heterogeneity. No significant difference was observed in balance function, risk of falling, and quality of life. Only a single available study reported a 70% adherence rate in the DHIs group. Subgroup analyses stratified by intervention duration revealed no significant intersubgroup differences for hip function (*χ*_1_^2^=0.1; *P*=.75) or functional independence (*χ*_1_^2^=2.93; *P*=.09). For hip function, the point estimate favored the 3 months subgroup (SMD 0.89, 95% CI 0.36-1.41; *I*^2^=7%; *P*=.41) over the <3 months subgroup. Conversely, for functional independence, the point estimate favored shorter intervention duration (SMD 0.67, 95% CI 0.12-1.23; *I*²=0%; *P*=.72).

**Conclusions:**

This review incorporates the latest randomized controlled trials and comprehensively assesses functional and psychological outcomes of DHIs in older patients with hip fractures, distinct from prior studies focusing solely on functional outcomes. While the 95% CI supports the potential of DHIs to improve hip function and functional independence, the wide 95% PI indicating substantial real-world response variability, which calls for cautious interpretation, informs the design of targeted DHI-based rehabilitation regimens, warranting further research into optimal techniques and dosages in clinical practice.

**Trial Registration:**

PROSPERO CRD42024626186; https://www.crd.york.ac.uk/PROSPERO/view/CRD42024626186

## Introduction

Hip fractures, particularly prevalent in older adults, present a significant and escalating public health challenge, characterized by well-documented morbidity, mortality, and health care burden [1-3]. By 2050, the annual global incidence of hip fractures is expected to reach 4.5 million [4], exerting ongoing pressure on existing health care and welfare systems [2,5]. With the general increase in human life expectancy, the systemic medical challenges posed by this condition are expected to intensify further. As per existing literature, the 30-day mortality rate after hip fractures can reach up to 10%, escalating further to 25% within the first year [6], while most surviving patients experience a significant decline in quality of life (QoL) [7]. Effective postsurgical rehabilitation is essential to mitigating the adverse outcomes of older adults with hip fractures, such as functional decline [8,9], psychological distress [10,11], and reduced QoL [9,12]. The majority of rehabilitation must now take place outside of hospitals due to shorter hospital stays brought on by a lack of health care resources and the need to increase hospital bed turnover rates [13].

Traditional rehabilitation programs face significant challenges in older adults, including limited access to health care services, low intervention adherence to prescribed rehabilitation programs, and insufficient psychological support. Adherence to rehabilitation programs remains a major issue, with many patients failing to complete recommended programs, thereby compromising functional recovery and increasing the risk of complications [14]. In addition, older adults may face specific barriers, such as limited mobility, geographic constraints, and resource shortages, which further reduce the feasibility and success of traditional rehabilitation methods [15-19]. Digital health interventions (DHIs) have emerged as a promising solution. DHIs, defined as the application of digital technologies in health care, leverage both conventional and innovative forms of information and communication technologies, such as mobile apps, telemedicine, wearable devices, and online platforms, to enhance health care delivery and patient engagement [20].

By bridging gaps in distance and accessibility, DHIs offer innovative solutions to overcome traditional health care barriers, particularly for populations with limited mobility, such as older patients with hip fractures [21]. Moreover, as digital technologies continue to advance, DHIs are evolving in both scope and sophistication, capable of providing more personalized, scalable, and effective approaches for the care of most patients whose rehabilitation training needs to be carried out outside the hospital [20,22]. DHIs have been used in the rehabilitation of older patients with hip fractures, and their potential effects have also been preliminarily demonstrated [13,21,23-26]. A study carried out by Gilboa et al [27] investigating a home-based tele-rehabilitation program, where occupational therapists remotely guided patients through personalized goal-setting and daily activity planning, demonstrated significant improvements in activities of daily living, health-related QoL, and mobility, while also reducing caregiver burden. In contrast to studies supporting DHIs, some researchers have argued that there are no or only minor effects detected in functional and psychological outcomes [28,29].

While the above findings highlight the potential impact of DHIs on functional recovery and psychological well-being in older adults with hip fractures, the evidence remains limited. Additionally, most studies focus primarily on functional recovery without adequately addressing psychological well-being and intervention adherence. Considering that the rehabilitation of older patients with hip fractures is an overall concept, it not only involves the recovery of function but also the restoration of psychological outcomes [30]. At the same time, studying the influence of intervention on intervention adherence is essential for the development of successful interventions in the rehabilitation of older patients with hip fractures [31]. The current research gaps underscore the need for a more comprehensive evaluation of DHIs in the context of the rehabilitation of hip fractures. With ongoing advancements in DHIs, a plethora of original studies focused on functional and psychological outcomes in older adults with hip fractures have been recently published. This makes it possible to review DHIs’ applications and guide future design and implementation of DHIs technology in clinical practice.

Thus, our systematic review aimed to comprehensively examine the effects of DHIs on functional and psychological outcomes, as well as intervention adherence in older adults with hip fractures. Compared with previous systematic reviews, this systematic review incorporates more recent research, includes a greater number of studies addressing psychological outcomes, and assesses the quality of the evidence [21].

## Methods

### Overview

This systematic review and meta-analysis was reported in accordance with the PRISMA (Preferred Reporting Items for Systematic Reviews and Meta-Analyses) 2020 statement (Multimedia Appendix 1) and PRISMA-S (Preferred Reporting Items for Systematic Reviews and Meta-Analyses literature search extension) to strengthen transparency (Multimedia Appendix 2) [32,33]. The review protocol was registered on the PROSPERO International Prospective Register of Systematic Reviews (CRD42024626186).

### Search Strategy

This systematic review was systematically searched in 9 scientific databases, including PubMed, Embase, CENTRAL, APA PsycINFO, Web of Science, PEDro, CNKI, WANFANG, and SinoMed. The literature search was updated by re-executing the predefined search string in the same 9 databases on November 13, 2025, and newly retrieved records were screened following the identical inclusion and exclusion criteria. Search strategies were originally designed by our research team and underwent a peer review process conducted by an independent medical doctor with extensive evidence-based medicine expertise, who verified the comprehensiveness of search terms, database selection, and eligibility criteria prior to the initiation of retrieval. No substantive modifications to the strategy were required following this review. No published search filters were used in our review. No search strategies from previously published literature reviews were adapted or reused for any part of our literature retrieval process.

Reference lists of eligible studies and cited studies (identified via Google Scholar) were manually screened to retrieve additional relevant literature. The ClinicalTrials.gov (United States Clinical Trials Registry) and the Chinese Clinical Trial Registry were searched for eligible studies.

Search engines and citation chasers were also screened to capture all relevant studies. For studies where the full text could not be obtained, corresponding authors were contacted to attempt to acquire the full text. Considering the availability of academic resources and the language proficiency of the research team, the included studies were limited to those published in English and Chinese with no date restriction. The full search strategies are available in Multimedia Appendix 3.

### Eligibility Criteria

We included the studies that related to (1) older adults (including patients aged 60 years or older) with hip fractures defined as any type of hip fractures, such as femoral neck, intertrochanteric, subtrochanteric fractures, and no specified type of hip fractures; (2) the intervention consisted of DHIs according to classifications of the World Health Organization (WHO) and must be provided or supported through DHIs for patients rather than trials targeting health care providers or other stakeholders [34]; (3) the primary outcome in this systematic review including functional outcomes (eg, hip function, walking capacity, and balance ability) and secondary outcomes including psychological outcomes (eg, depression and QoL) and intervention adherence; (5) the comparator was usual care, control group or no intervention; and (6) the study design limited to randomized controlled trials (RCTs). The following studies were excluded: (1) patients enrolled in the emergency department but not admitted to the hospital, (2) patients discharged from the hospital to a setting other than home, (3) studies involving DHIs that did not directly intervene with patients (including database management), and (4) studies with inaccessible full text or insufficient data for evaluating the characteristics of DHIs or quantitative synthesis.

### Study Selection and Data Extraction

After automatic and manual removal of duplicate records using the reference management software EndNote 21 (Clarivate Analytics), 2 review authors (WF and QZ) independently screened titles and abstracts and then full texts against predefined eligibility criteria. A senior author (QL) resolved conflicts.

Two authors (WF and QZ) independently but in parallel collected relevant data in a standardized data collection sheet, including but not limited to characteristics of the included studies, such as the authors, publication year, country, and study design, as well as participant characteristics, including sample size, sex ratio, average age, and the details of the intervention and control groups.

For studies with multiple intervention arms, data from the relevant arms were combined to create a single pairwise comparison, as recommended in the Cochrane Handbook, to avoid double-counting participants in the control group. For studies reporting multiple time points for the same outcome, data at the end of the intervention were extracted for meta-analysis. Any discrepancies between groups were resolved through discussion and consensus, and when necessary, a senior reviewer (QL) was consulted to adjudicate unresolved differences.

### Risk-of-Bias and Grading of Recommendations Assessment, Development, and Evaluation

Risk of bias in each study was assessed independently by 2 authors (WF and QZ). The RCTs were evaluated using the Cochrane Risk of Bias tool 2.0 (Cochrane Collaboration), which assesses random sequence generation, allocation concealment, blinding of participants and personnel, blinding of outcome assessment, incomplete outcome data, selective reporting, and other bias [35]. The reviewers made a judgment of “yes” (low bias), “no” (high bias), or “unclear” (lack of relevant information or uncertainty of bias) for each item. Included studies were also subjected to scoring for certainty of evidence using GRADE (Grading of Recommendations, Assessment, Development and Evaluation) criteria independently by 2 authors (WF and QZ). Any discrepancies between groups were resolved through discussion and consensus, and when necessary, a third reviewer (QL) was consulted to adjudicate unresolved differences.

### Meta-Analysis Methods

R (version 4.0.3; R Foundation for Statistical Computing) was used to conduct the meta-analyses. The data analysis was based on postintervention means and SDs, with conversion performed using established formulas when only change values were reported in the original studies [36]. The effect measures were estimated through the standardized mean difference (SMD) based on Hedges *g*, along with the 95% CI. To assess statistical heterogeneity, τ² was calculated. The Cochran Q test and *I²* were also derived, which together quantify the extent and significance of the observed heterogeneity. Considering the expected significant heterogeneity among studies, we adopted a random-effects model to combine the effect sizes. The estimation of the CI was performed by the HKSJ (Hartung-Knapp-Sidik-Jonkman) method. For outcomes with enough studies (n3), we calculated 95% prediction intervals (PIs) to estimate the range of true effects in future settings. Forest plots are used to present the results of the merger.

Subgroup analyses were conducted to explore sources of heterogeneity, stratified by (1) type of DHIs (mobile health [mHealth]: telephone calls, text messages, and apps; eHealth: internet based, websites, and emails; or digital device: wearables and Bluetooth-connected devices) [37]; and (2) the duration of intervention (<3 months vs ≥3 months). The duration threshold was chosen based on common clinical rehabilitation milestones. If a prespecified subgroup analysis is not feasible due to a small number (<3 studies) of included studies, we will use narrative synthesis to summarize the included studies.

In addition, sensitivity analyses were conducted by sequentially excluding one study at a time to assess the reliability and stability of the main outcome (hip function). A funnel plot and Egger test were used to assess the small study effect. All tests were 2-tailed with a significance level of α=.05.

## Results

### Search Findings

**Figure 1:**
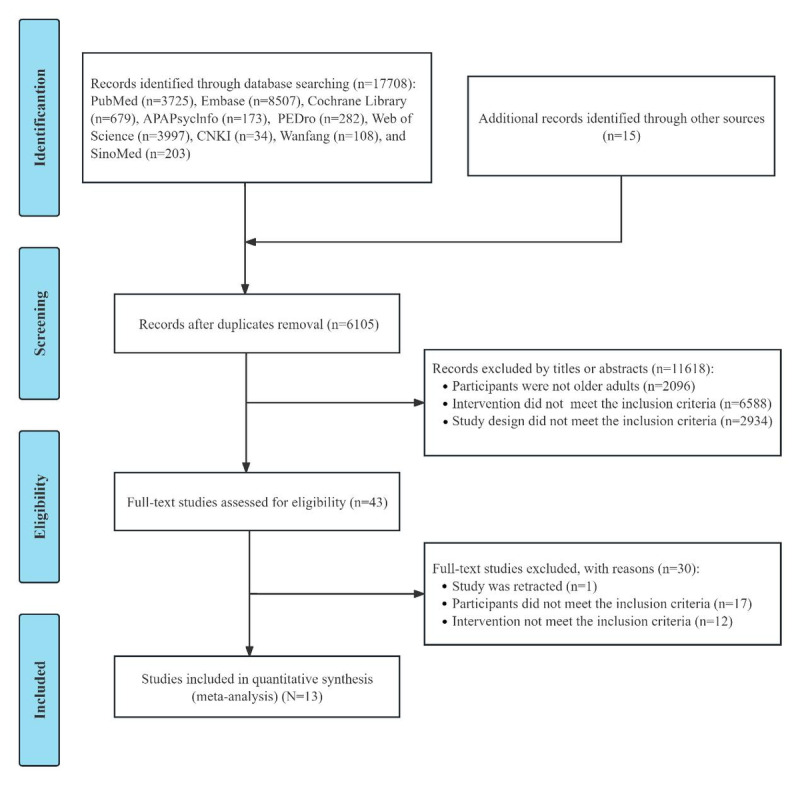
Flowchart of the study selection process.

The initial database search yielded 17,708 records, and an additional 15 records were identified through other sources, resulting in a total of 17,723 records. After removing 6105 duplicates, 11,618 records were excluded by title and abstract screening. Subsequently, the remaining 43 studies were screened for full text. Finally, a subset of 13 studies [29,38-49] was included. The PRISMA flow diagram is shown in Figure 1.

### Characteristics of Included Studies

This systematic review included 13 studies [29,38-49] with 737 participants in total and published between 2015 and 2025 (Table 1). Eight studies were implemented in China [29,38-44], and one each in Israel [45], Sweden [46], Spain [47], Thailand [48], and Canada [49]. The age of participants ranged from 65.7 (SD 7.8) to 86.0 (SD 16.0) years, and the percentage of males ranged from 27.2% (38/138) to 80.6% (25/31). The intervention lasted between 3 weeks and 4 months, and the duration of follow-up was from 3 weeks to 9 months.

**Table 1:** Characteristics of included studies.

Study, year, country, and study design	Sample size (IG^a^:CG^b^); sex (M^c^:F^d^); age (IG:CG)	Intervention type and provider	Contents of the intervention group	Control group	Outcome and outcome measurement tools
Kalron et al [45], 2018, Israel: RCT^e^ (FS^f^), Blind (participants, investigators)	32 (15:17); 20:12; 65.7(7.8):67.3(9.5)	mHealth^g^ (app): physiotherapist	Video lesson (includes health education, rehabilitation training standards, and key points of training movements) and progressive rehabilitation training. Frequency: 5 times per week. Duration: 4 months.	Regular manage	Balance function and risk of falling: TUG^h^
Li et al [38], 2022, China, Hong Kong: RCT, Blind (outcome assessors)	31 (15:16); 25:6; 76.5 (8.6):82.1 (9.7)	eHealth (internet-based): physiotherapist	Telerehabilitation was delivered through the Caspar Health e-system (CASPAR Health) to initiate falls prevention information, coaching to remain active, problem-solving skills, mobility goal setting, and advice to help participants maintain and increase their prescribed home exercises. Frequency: personalized. Duration: 8 weeks.	Usual care	Functional independence: BI^i^ Balance function and risk of falling: TUG
Howell et al [46], 2023, Sweden: RCT (FS), Blind (participants)	21 (10:11); 15:6; 86.0 (16.0):83.0 (13.0)	Digital device (wearables): physiotherapist	Participant wore a wearable device to measure body position and movements; the therapist receives feedback and can ensure that the rehabilitation program is tailored to the needs of the individual. Frequency: no mention. Duration: 4 weeks.	Standard rehabilitation	Functional independence: BI Balance function and risk of falling: FBG^j^ QoL^k^: EQ-5D
Langford et al [49], 2015, Canada: RCT, Blind (investigators, outcome assessors)	30 (15:15); 19:11; 83.0 (8.0):82.0 (10.0)	mHealth (app): physician	Exercises relating to movement, strength of the lower limbs, and balance performance were included by using a mobile app. Frequency: personalized. Duration: 3 weeks.	Usual care	QoL: EQ-5D
Cheng et al [29], 2022, China, Hong Kong: RCT, Open label	39 (19:20); 20:12; 75.8 (7.2):79.0 (8.8)	mHealth (app): physiotherapist	Exercises with embedded videos in the app, receive a home-based rehabilitation program, and caregiver education. Frequency: once per day. Duration: 2 months.	Standard rehabilitation	Functional independence: MFAC^l^
Prieto-Moreno et al [47], 2024, Spain and Belgium: RCT, Blind (outcome assessors)	105 (51:54); 30:75; 79.6 (7.1):80.1 (7.7)	eHealth: (internet-based); no mention	Health knowledge, education, and telerehabilitation guidance, including physical exercise and an occupational therapy session. Frequency: twice a week. Duration: 12 weeks.	Routine rehabilitation	Functional independence: FIM^m^ QoL: EQ-5D
Li [44], 2024, China: RCT, No mention	80 (38:42); 47:33; 68.5 (5.6):69.4 (5.0)	mHealth (app): physician	Remote rehabilitation guidance to the patients and their family caregivers via WeChat (Tencent) video, answered the patients’ questions, and guided functional exercises. Frequency: once each in the second, fourth, and eighth weeks. Duration: 12 weeks.	Routine health education	Hip Function: HHS^n^ QoL: SF-12^o^
Zhang [42], 2024, China: RCT, Blind (outcome assessors)	61 (30:31); 36:25; 69.0 (4.5):70.0 (7.4)	mHealth (app): physiotherapist	The experimental group provided exercise guidance and training feedback to the subjects through a mobile app (Joymotion software, Shanghai Fudong Medical Management Co, Ltd), and conducted real-time 2-way audio and video interactive consultations with physical therapists. Frequency: 5 times a week. Duration: 4 weeks.	Routine rehabilitation	Hip function: HOOS^p^ QoL: SF-12
Gao et al [39], 2021 China: RCT (No mention)	80 (40:40); 30:50; 77.8(5.9):78.1(5.6)	mHealth (app): physician	Doctors transmitted text, pictures, voice, and video to the group to guide and urge patients to perform rehabilitation exercises through WeChat. For special patients, doctors would provide personalized and targeted guidance. Frequency: no mention. Duration: 2 months.	Routine rehabilitation	Hip function: HHS
Zhang (1) [40], 2021, China: RCT, Blind (participants, outcome assessors)	51 (27:24); 18:33; 77.0 (7.9):75.2 (7.7)	eHealth (internet-based): physiotherapist	The experimental group adopted the “Family-Oriented Postoperative Rehabilitation Management System for Elderly Hip Fractures” developed by Tianjin Hospital of Tianjin, which was used for remote home rehabilitation. This system is based on technologies such as Internet Plus, the Internet of Things, cloud computing, mobile Internet, and big data. It adopts the approach of “integrated application of technologies and rapid combination of modules” to establish a family rehabilitation treatment platform. Frequency: no mention. Duration: 3 months.	Usual care	Hip function: HHS Functional independence: FIM Balance function and risk of falling: TUG QoL: SF-36^q^
Zhang (2) [41], 2021, China: RCT (No mention)	36 (18:18); 15:21; 72.4 (8.4):68.8 (12.5)	mHealth (app): rehabilitation team	Participants received remote rehabilitation based on network media support, mainly using 2 social software programs, WeChat and TikTok (ByteDance). First, establish a WeChat group composed of rehabilitation physicians, surgeons, physical therapists, head nurses, patients, and their families. Make full use of their spare time to provide real-time rehabilitation guidance to patients and answer their questions and their families’ questions. Frequency: no mention. Duration: 10 weeks.	Usual care	Hip Function: HHS QoL: SF-36
Dajpratham et al [48], 2025, Thailand: RCT, Blind (investigators)	33 (16:17); 6:27; 75.1 (9.5):78.3 (7.7)	mHealth (app): physiotherapist	Real-time videoconference exercises with a physiotherapist via the LINE app. Frequency: 3 times per week, with each session lasting approximately 45 minutes. Duration: 12 weeks.	Usual care	Balance function and risk of falling: SPPB^r^ Intervention adherence: percentage format
Shui [43], 2025, China: RCT (No mention)	138 (69:69); 38:100; 76.0 (5.7):75.2 (5.6)	mHealth (app): rehabilitation team	Family remote rehabilitation program training: the attending physician, rehabilitation physician, and responsible nurse established an online communication group through the WeChat platform to strengthen patient management and guide the training content. Frequency: 3 times per month. Duration: 3 months.	Usual care	Hip function: HHS Functional independence: FIM QoL: EQ-5D

^a^IG: intervention group.

^b^CG: control group.

^c^M: male.

^d^F: female.

^e^RCT: randomized controlled trial.

^f^FS: feasibility study.

^g^mHealth: mobile health.

^h^TUG: Timed Up and Go test.

^i^BI: Barthel Index.

^j^FBG: Functional Balance for Geriatric Patients.

^k^QoL: quality of life.

^l^MFAC: Modified Functional Ambulatory Category.

^m^FIM: Functional Independence Measure.

^n^HHS: Harris Hip Score.

^o^SF-12: Short Form–12 Health Survey.

^p^HOOS: Hip Disability and Osteoarthritis Outcome Score.

^q^SF-36: Medical Outcomes Study 36-Item Short Form Health Survey.

^r^SPPB: Short Physical Performance Battery.

In the 13 included studies [29,38-49], the type of DHIs could be divided into 3 categories, which included mHealth (n=9 [29,39,41-45,48,49]), eHealth (n=3 [38,40,47]), and digital device (n=1 [46]). The involvement of health care providers in DHIs varied across studies: a rehabilitation team was involved in 2 studies [41,43], physiotherapists in 7 studies [29,38,40,42,45,46,48], physicians in 3 studies [39,44,49], and 1 study [47] did not specify the intervention provider.

Of the 13 included trials [29,38-49], outcomes of our interest included hip function (6 studies [39-44]), functional independence (6 studies [29,38,40,43,46,47]), balance function and risk of falling (5 studies [38,40,45,46,48]), QoL (8 studies [40-44,46,47,49]), and intervention adherence (1 study [48]). Although the instruments used for outcome evaluation varied across studies, all were valid scales, and the process of data collection was carried out. Six studies [39-44] that reported hip function were assessed using the Harris Hip Score (HHS) [39-41,43,44] and Hip Disability and Osteoarthritis Outcome Score (HOOS) [42]. Six trials [29,38,40,43,46,47] evaluated functional independence using the Functional Independence Measure (FIM) [40,43,47], the Modified Functional Ambulatory Category (MFAC) [29], and the Barthel index [38,46]. Instruments used to evaluate balance function and risk of falling included the Timed Up and Go test (TUG) [38,40,45], Short Physical Performance Battery (SPPB) [48], and Functional Balance for Geriatric Patients (FBG) [46]. Eight studies [40-44,46,47,49] that reported QoL were assessed using the Medical Outcomes Study (MOS) 36-Item Short Form Health Survey (SF-36) [40,41], the EQ-5D scale [43,46,47,49], and the Short Form-12 Health Survey (SF-12) [42,44]. Intervention adherence was described using the percentage format in a single study [48].

### Risk of Bias

**Figure 2:**
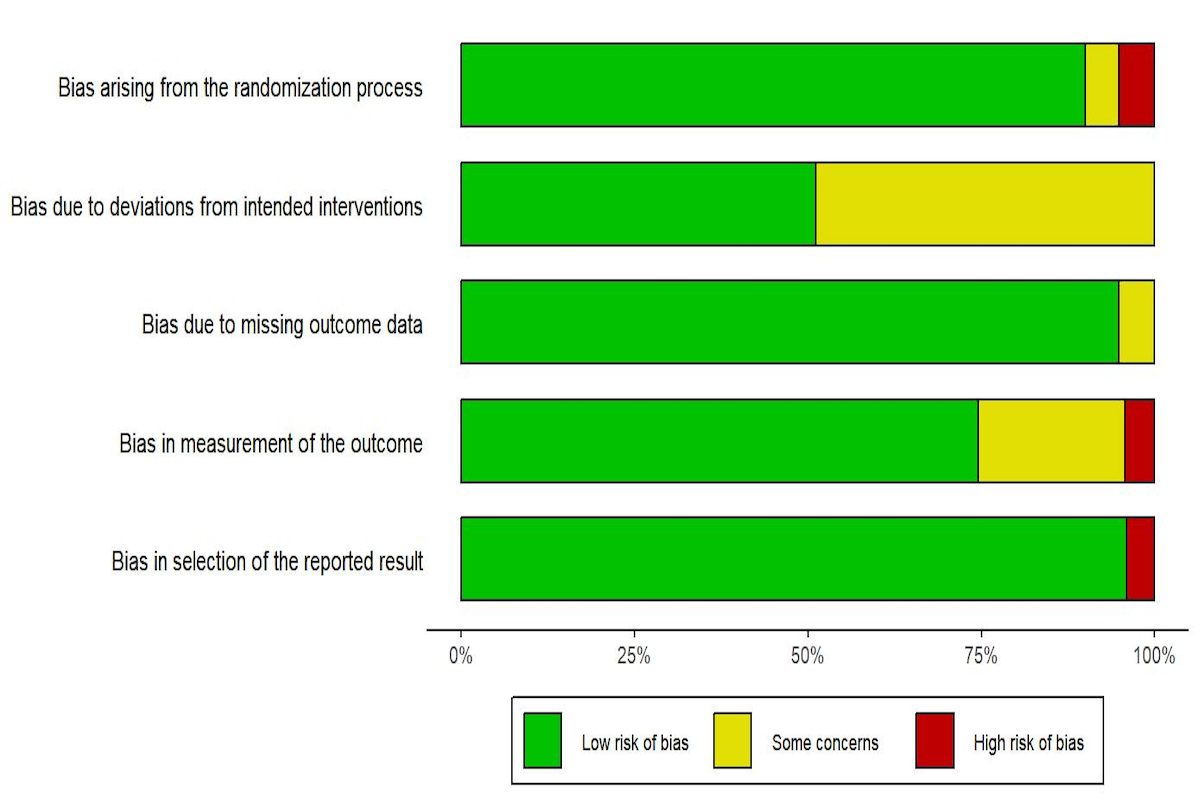
Risk of bias.

Overall, 5 studies [40,42,45,47,48] had low risk of bias, and 5 studies [38,39,41,43,44] had some concerns. Another 3 studies [29,46,49] had a high risk of bias, 1 study [29] had a high risk in terms of the randomization process, 1 study [46] had a high risk in terms of the measurement of the outcome, and 1 study [49] had a high risk in terms of the selection of the reported result. Detailed results are presented in Figure 2.

### Certainty of Evidence

The GRADE assessment scores ranged from low to moderate (Table 2). Evidence was downgraded if (1) the risk of bias was apparent; (2) inconsistency was demonstrated, with *I*^2^>75%; (3) there was indirectness in participants or comparators; (4) there was evidence of imprecision; or (5) there was publication bias. An overall GRADE rating was agreed upon (WF, QZ, and QL) for each outcome at 4 levels, including very low, low, moderate, and high.

**Table 2:** GRADE (Grading of Recommendations, Assessment, Development and Evaluation) summary of findings.

Outcomes	Number of participants (studies)	Quality of evidence (GRADE^a^)	Relative effect (SMD^b^, 95% CI)	Anticipated absolute effect	
				Control group, mean (SD)	DHIs^c^ group, mean (95% CI)
Hip function	435 (6 RCTs^d^)	( 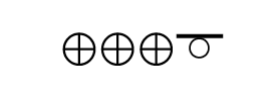 ) Moderate^e,f^ (due to imprecision)	0.80 (0.33-1.26)	HHS^g^: 56.71 (14.71)	68.48 (61.56-75.24)
Functional independence	369 (6 RCTs)	( 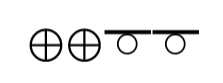 ) Low^e,f^ (due to inconsistency, imprecision)	1.23 (0.34-2.11)	FIM^h^: 82.67 (5.69)	89.67 (84.60-94.68)
Balance function and risk of fall	163 (5 RCTs)	( 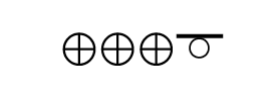 ) Moderate^f^ (due to imprecision)	–0.09 (–0.99 to 0.81)	TUG^i^: 34.16 (15.07)	32.80 (19.24-46.37)
Quality of life	501 (8 RCTs)	( 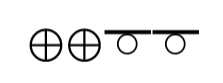 ) Low^e,f^ (due to inconsistency, imprecision)	0.65 (–0.21 to 1.50)	SF-36^j^: 56.71 (14.71)	66.27 (53.62-78.78)
Intervention adherence	33 (1 RCT)	—^k^	—	—	—

^a^GRADE: Grading of Recommendations, Assessment, Development, and Evaluation.

^b^SMD: standard mean difference.

^c^DHI: digital health intervention.

^d^RCT: randomized controlled trial.

^e^Inconsistency.

^f^Imprecision.

^g^HHS: Harris Hip Score.

^h^FIM: Functional Independence Measure.

^i^TUG: Timed Up and Go.

^j^SF-36: MOS 36-Item Short Form Health Survey.

^k^Not available.

### Findings of Meta-Analysis

#### Effects of DHIs on Hip Function

**Figure 3:**
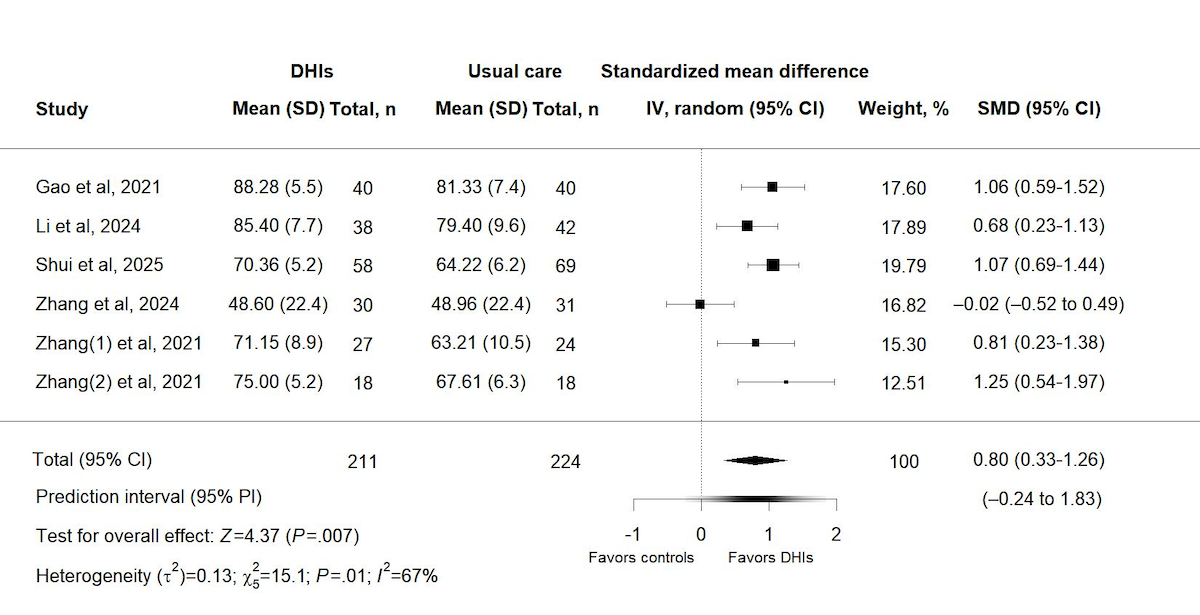
Forest plot for the effect of digital health interventions on hip function [39-44]. DHI: digital health intervention; SMD: standardized mean difference.

As shown in Figure 3, 6 trials [39-44] with 435 patients were eligible for pooling for the analysis of hip function outcome. The pooled results from the random-effects model indicated that DHIs significantly improved hip function compared to the control group, with an SMD of 0.80 (95% CI 0.33-1.26; *P*=.007). The 95% PI (–0.24 to 1.83) further quantified the range of potential treatment effects across individual patients and clinical settings. Although pooled results suggest that DHIs yield a favorable average effect, some patients or subgroups might experience negligible or even marginal negative responses to the intervention, which aligns with the substantial heterogeneity observed (*χ*_5_^2^=15.1; *P*=.01; *τ*²=0.13; *I*²=67%).

#### Effects of DHIs on Functional Independence

**Figure 4:**
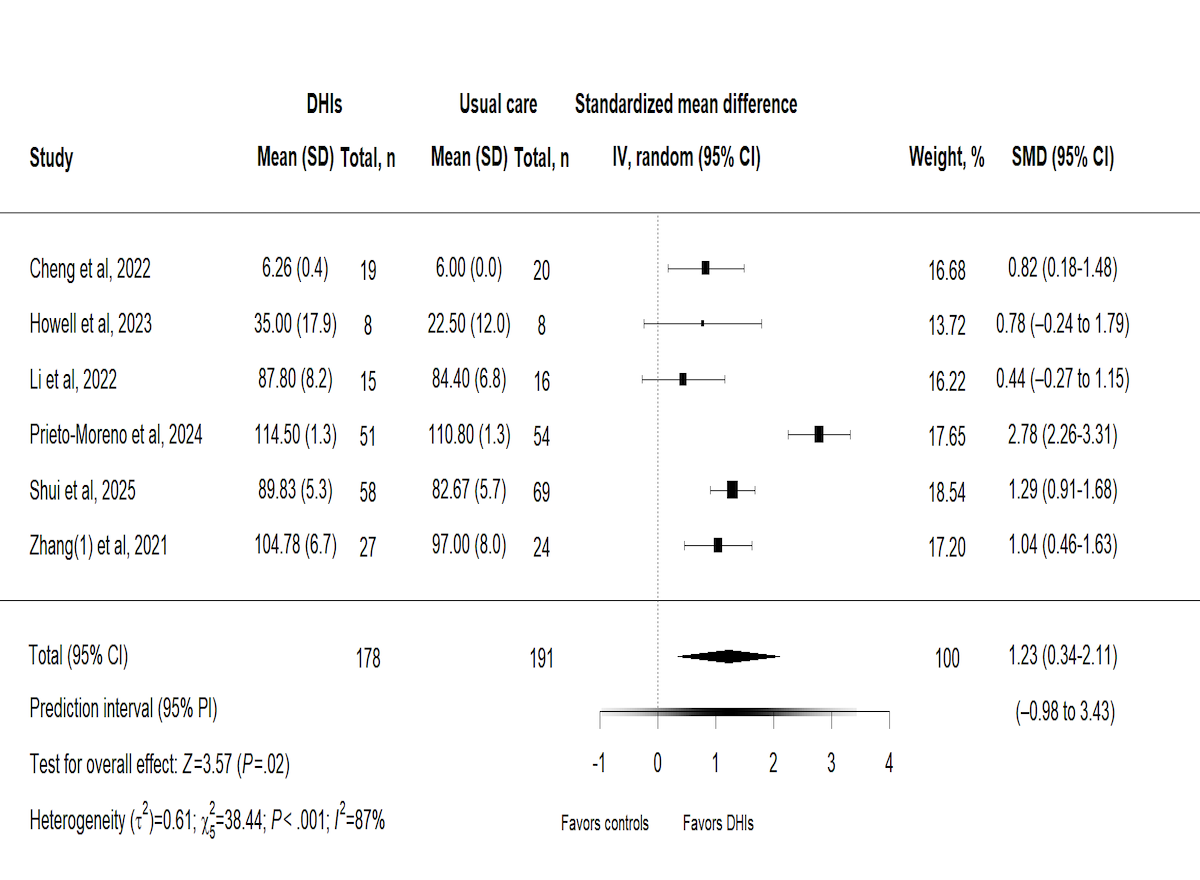
Forest plot for the effect of digital health interventions on functional independence [29,38,40,43,46,47]. DHI: digital health intervention; SMD: standardized mean difference.

The effects of DHIs on functional independence were measured in 6 trials [29,38,40,43,46,47] involving 369 participants (Figure 4). Based on a random-effects model, pooled results showed a statistically significant beneficial effect of DHIs on enhancing functional independence in older patients with hip fractures, with an SMD of 1.23 (95% CI 0.34-2.11; *P*=.02). In stark contrast, the 95% PI (–0.98 to 3.34) reflects the wide spectrum of individual responses to DHIs, indicating that while DHIs yield a favorable mean effect, some patients may experience no improvement or even a slight decline in functional independence, whereas others could achieve marked benefits. This pronounced variability aligns with the extremely high heterogeneity (*χ*_5_^2^=39.7; *P*=.02; *τ*²=0.62; *I*^2^=87%) observed in the meta-analysis, highlighting the critical role of patient-specific and trial-related factors in modulating the effects of DHIs.

#### Effects of DHIs on Balance Function and Risk of Falling

**Figure 5:**
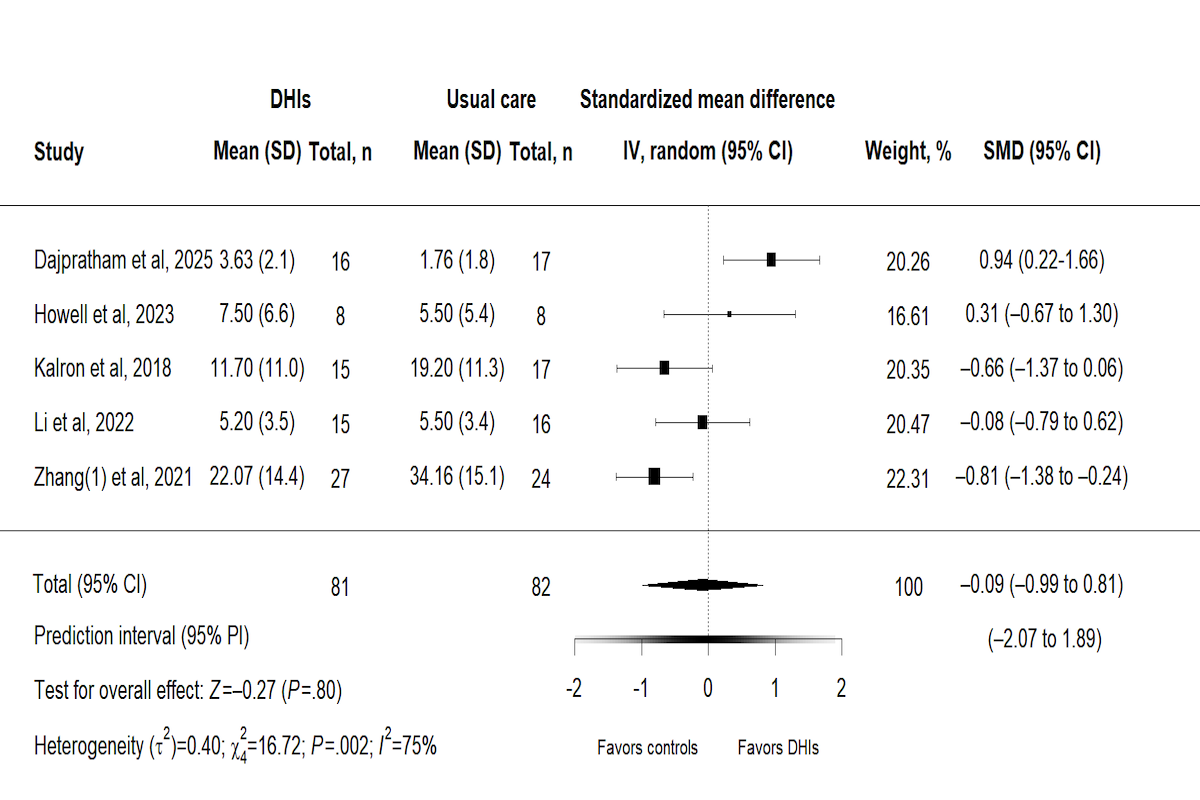
Forest plot for the effect of digital health interventions on balance function and risk of falling [38,40,45,46,48]. DHI: digital health intervention; SMD: standardized mean difference.

In terms of balance function and risk of falling, it was measured in 5 trials [38,40,45,46,48] involving 163 participants. Based on a random-effects model, the results showed that compared to usual care, DHIs had no significant effects on improving balance function and reducing risk of falling (SMD –0.09, 95% CI –0.99 to 0.81; *P*=.80). The 95% PI (–2.07 to 1.89) was further estimated to delineate the variability of individual treatment responses and indicated that while the mean effect is negligible, some patients might experience meaningful improvements in balance function with DHIs, whereas others could exhibit worse outcomes. This pronounced variability in individual responses corresponds well with the high heterogeneity (*χ*_4_^2^*=*16.7; *P*=.002*;* *τ*^2^=0.40; *I*^2^=75%; Figure 5).

#### Effects of DHIs on Quality of Life

**Figure 6:**
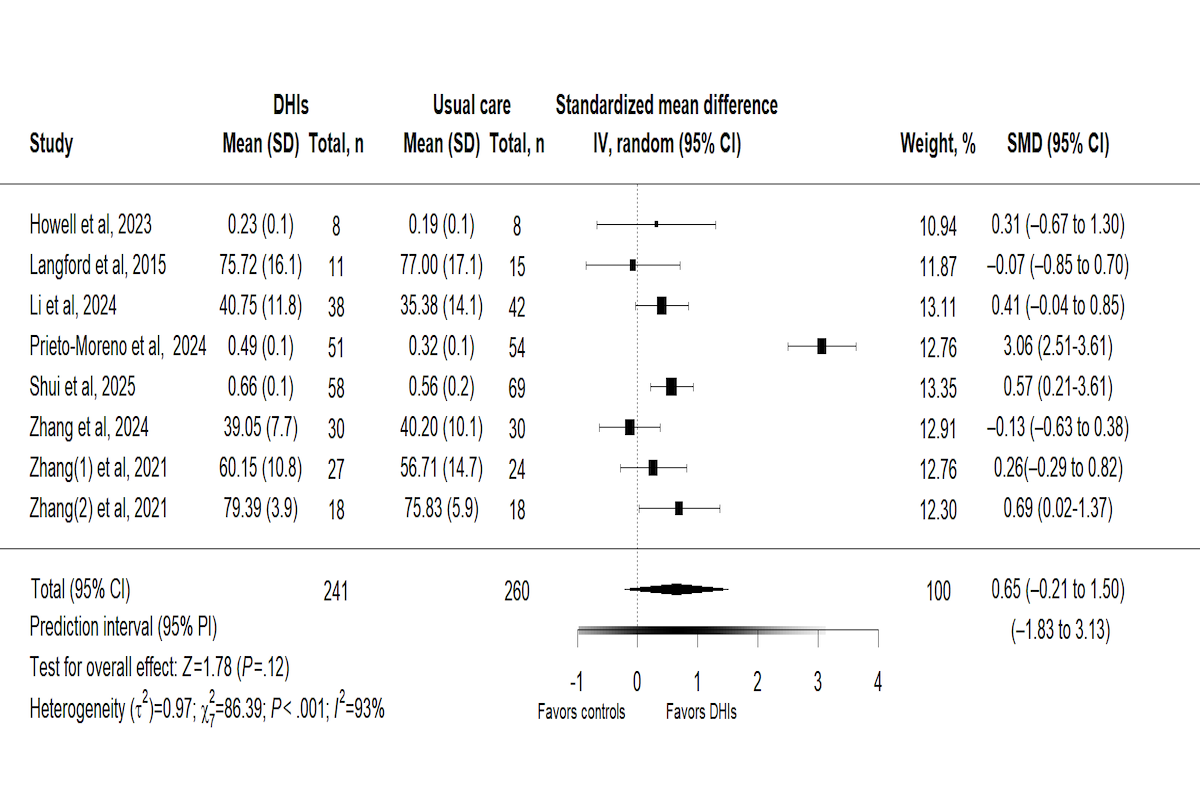
Forest plot for the effect of digital health interventions on quality of life [46,49,44,47,43,42,41,40]. DHI: digital health intervention; SMD: standardized mean difference.

The effect of DHIs on QoL was measured in 8 trials [40-44,46,47,49] involving 501 participants. This meta-analysis adopted a random-effects model for data pooling. The overall results indicated that there was no statistically significant difference in QoL between groups, with an SMD of 0.65 (95% CI –0.21 to 1.50; *P*=.12). The 95% PI (–1.83 to 3.13) was further calculated, and substantial statistical heterogeneity was observed among the included trials (*χ*_7_^2^=89.5; *P*<.001; *τ^2^*=0.97; *I*^2^=93%; Figure 6).

#### Effects of DHIs on Intervention Adherence

Due to the limited reporting of intervention adherence data across studies, a meta-analysis was not possible; only a single available study reported a 70% intervention adherence rate in the intervention group [48].

#### Subgroup Analyses

##### Subgroup Analysis by Type of Intervention

A subgroup analysis based on the type of DHIs was planned. However, the number of studies in each predefined intervention category (mHealth; n=9 [29,39,41-45,48,49], eHealth; n=3 [38,40,47], and digital device; n=1 [46]) fell below the threshold of 3 for all outcomes, and the planned subgroup analysis based on the type of DHIs was therefore forfeited due to the inability to provide meaningful quantitative estimates.

##### Subgroup Analysis by Intervention Duration

**Figure 7:**
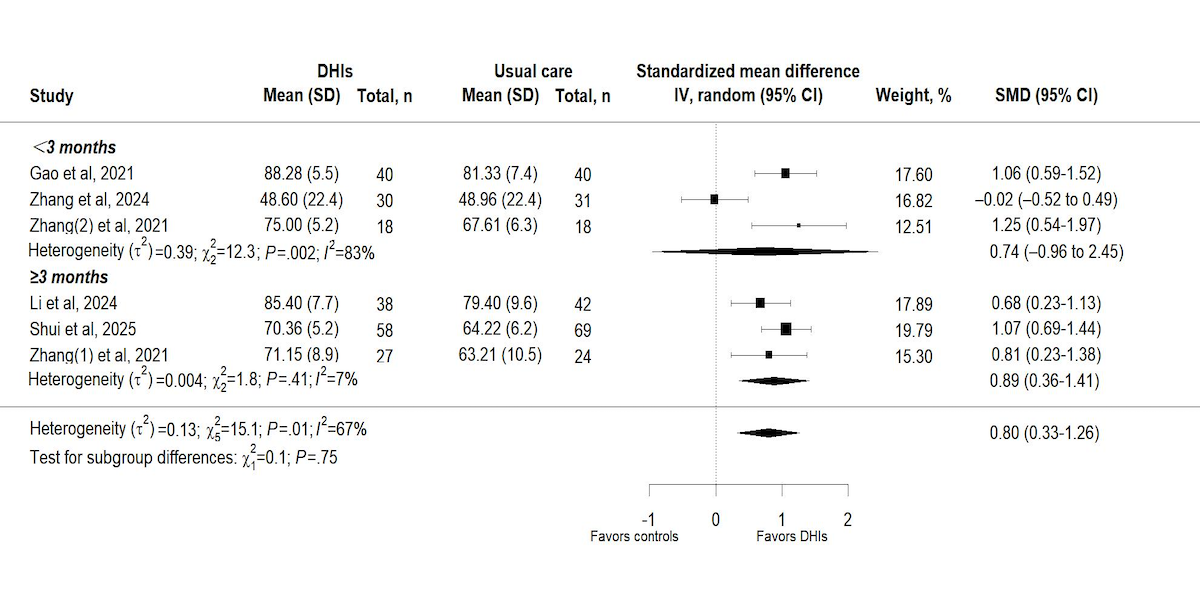
Subgroup analysis on hip function [39,42,41,44,43,40]. DHI: digital health intervention; RE: Random effect; SMD: standardized mean difference.

**Figure 8:**
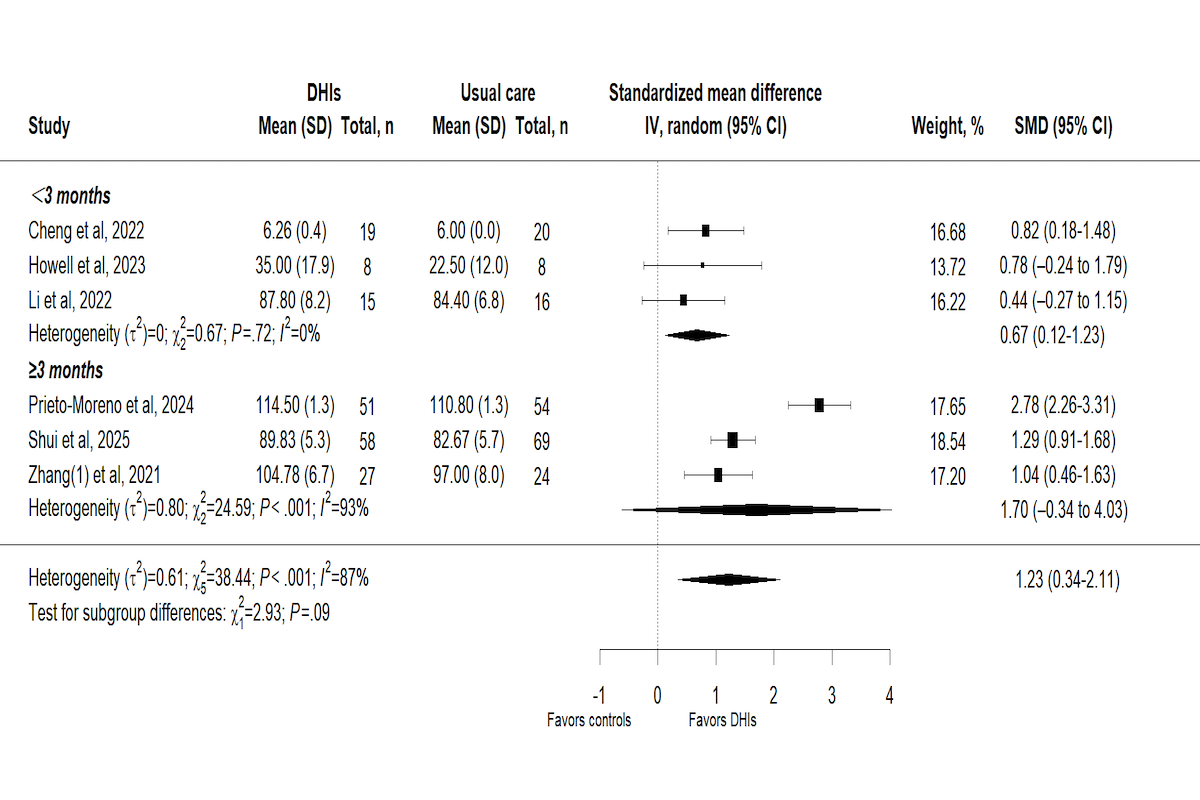
Subgroup analysis on functional independence [29,38,40,43,46,47]. DHI: digital health intervention; SMD: standardized mean difference.

Subgroup analysis was conducted based on the intervention duration (<3 months vs ≥3 months) to explore the effects of DHIs on hip function. The subgroup analysis for hip function revealed no statistically significant difference between subgroups (χ_1_^2^=0.1; *P*=.75), with interventions lasting ≥3 months showing a greater effect (SMD 0.89, 95% CI 0.36-1.41; *I*²=7%; *P=*.41) compared to those lasting <3 months (Figure 7). The test for subgroup differences in functional independence suggested a marginally significant variation in effects (*χ*_1_^2^=2.93; *P*=.09), indicating that intervention duration may be a potential contributor to the overall heterogeneity of the meta-analysis, albeit not reaching formal statistical significance, while the point estimate still favored shorter intervention duration (SMD 0.67, 95% CI 0.12-1.23; *I*²=0%; *P=*.72; Figure 8).

#### Small-Study Effects and Sensitivity Analysis

The funnel plot for hip function underlying the meta-analyses was symmetrical, and the Egger test was conducted to assess potential small-study effects, and no statistically significant evidence of such effects was identified in this meta-analysis (*z*-score=0.84; *P*=.40; Multimedia Appendix 4). Sensitivity analysis, performed by sequentially removing each study, confirmed that no single study unduly influenced the overall pooled estimate for hip function, demonstrating the robustness of this finding (Multimedia Appendix 4).

## Discussion

### Principal Findings

This meta-analysis confirms that DHIs confer statistically reliable average benefits for hip function and functional independence in older adults with hip fractures, whereas their effects on balance and QoL remain inconclusive. A wide 95% PI, however, indicates substantial real-world variability in individual DHI responses, restricting broad clinical deployment. Subgroup analyses also reflect this inconsistency—with intervention duration providing partial explanatory power—but the limited number of included studies necessitates further research to elucidate the underlying sources of such heterogeneity. Additionally, intervention adherence was reported in only 1 study [48], precluding quantitative assessment of DHI-related effects on this outcome. These results underscore that the potential of DHIs to promote functional and psychological recovery in this population requires further validation.

Low-to-moderate-certainty evidence, as determined by the GRADE assessment, suggests that DHIs are superior to control conditions in improving hip function and functional independence among older adults with hip fractures. This finding aligns with previous research supporting the role of telerehabilitation interventions in enhancing physical function following joint surgery and has suggested that this is related to the ability of DHIs to provide effective communication and feedback, as well as an educational component [21,50,51]. Health education during the postoperative rehabilitation stage is crucial for patients’ recovery. Postoperative patients need to receive numerous recommendations, including early ambulation, exercise and rehabilitation programs, preventing postoperative complications, modifying home hazards, and taking nutrition supplements [21,52]. DHIs mostly adopt mobile apps as the mode of delivery, which can provide real-time or asynchronous communication and feedback. It can break down the boundaries of time and space, providing an effective communication platform between physiotherapists and patients, which is conducive to accurately transmitting rehabilitation knowledge and ultimately achieving positive functional recovery effects [53,54].

In addition, most DHIs systems incorporate exercise training, allowing physiotherapists to provide exercise guidance through voice, video, and other means, while receiving feedback from patients [55]. This helps patients correct improper movements promptly and ensures training quality [56]. Studies have shown that appropriate exercise after orthopedic surgery can counteract muscle weakness and functional disorders that may be caused by limited limb usage and also improve the range of joint motion, which is conducive to the recovery of the hip joint and physical function [21,57]. Currently, DHIs predominantly use combined exercises, such as resistance or strength training paired with balance training, to maximize functional improvement. Resistance training and strength training can not only enhance muscle strength but also lead to the upregulation of bone turnover and subsequent bone deposition, which has a positive regulatory effect on bone health [58-60]. Balance training is expected to improve balance function and prevent falls.

However, it is critical to emphasize that the effects of DHIs exhibited substantial real-world variability. For hip function, the 95% PI spanned from marginally negative to large positive treatment responses, which quantifies the true heterogeneity across populations and settings far more pragmatically than *I*^2^ alone. For functional independence, the 95% PI covered an even broader range of individual patient responses, consistent with extremely high between-study heterogeneity. This pattern indicates inconsistent DHIs' self-efficacy across studies and patient subgroups in real-world settings. In addition, variations in study design (eg, differences in individual characteristics, DHIs delivery modes, and control group interventions) may have introduced bias, potentially confounding the pooled effect estimates. The low-to-moderate certainty of evidence reflects limitations, including imprecision in effect estimates and inconsistency across trials. Collectively, these factors mandate cautious clinical translation of the findings.

Moderate-certainty evidence suggested that the effects of DHIs on balance function and fall risk were uncertain, which contrasts with previous studies. Previous studies have reported the positive effects of DHIs on improving balance ability in populations, such as older adults and patients with neurological diseases [61-63]. A study investigating the mechanism of balance improvement in older adults after balance training found that balance training can increase the duration of cocontraction of the soleus or tibialis anterior, which can enhance joint stability and thereby improve the balance control ability of older adults [64]. However, the effects of balanced training also seem to have a dose-response relationship [65]. Earlier studies showed that a high practice intensity and an increased session frequency led to significant retention over the longer term [66,67]. However, increasing the intensity, frequency, and duration of such protocols could also have disadvantages, such as activity-induced fatigue and reduced participation [68]. It has also been found that the plasticity of the human brain provides physiological preparation for the restoration of balance function, but this still requires long-term persistence [69]. Therefore, the fact that no significant improvement in balance function was observed in this systematic review might be due to the lack of a suitable balance training program for older patients with hip fractures, or the duration of intervention was not sufficient to restore balance function.

It should also be noted that the wide 95% PI for balance function and fall risk reflects pronounced variability in individual patient responses, aligning with the high between-study heterogeneity observed. Additionally, many trials lacked standardized assessment tools for these outcomes and failed to implement outcome assessor blinding, and these methodological limitations likely introduced bias, confounded pooled effect precision, and contributed to the observed heterogeneity. The evidence for effects of DHIs on balance function and fall risk is rated as moderate certainty, owing to imprecise effect sizes. Collectively, these factors indicate that the null average effect should not be interpreted as definitive proof of DHIs’ ineffectiveness, and instead, the broad PI underscores the potential for subgroup-specific benefits or harms that aggregate analyses cannot capture.

Contrary to our expectations, low-certainty evidence showed no effect of DHIs on QoL. Molina-Garcia et al [70] indicated that telerehabilitation interventions can significantly improve QoL in patients with musculoskeletal disorders. This difference may be attributed to the fact that Molina-Garcia et al [70] focused on a broader range of musculoskeletal diseases, while our meta-analysis specifically targeted patients with hip fractures—a population typically characterized by advanced age, multiple diseases, and slow recovery [71]. Additionally, the intensity and duration of DHIs conducted in existing studies may not be sufficient to overcome the profound physical and psychological challenges associated with this injury and restore the QoL of older patients to the level before the fracture [12,72]. A cohort study also showed that social support and pain while walking can predict QoL among older adults 3 months after discharge from rehabilitation following a surgical hip fracture repair [73]. It indicates that the failure to effectively address pain may indirectly lead to a lack of statistically significant improvement in QoL outcomes. Therefore, in order to improve the psychological outcomes of older patients with hip fractures, it is best to integrate pain management and social support measures into DHIs.

The wide 95% PI also reflects pronounced variability in QoL in individual patient responses to DHIs, which aligns closely with the extremely high between-study heterogeneity observed. Included trials lacked a uniform QoL instrument and failed to account for confounding factors, such as baseline mental health status and social support levels, introducing methodological bias that may have inflated heterogeneity and compromised the precision of pooled estimates. Consequently, the evidence for effects of DHIs on QoL is rated as low certainty, constrained by imprecise effect sizes and inconsistent reporting of long-term QoL trajectories. Collectively, these findings indicate that the null average effect should not be equated with a lack of DHIs’ efficacy, and instead, the broad PI highlights the potential for substantial subgroup-specific benefits or null effects that aggregate analyses cannot fully delineate.

Although a formal meta-analysis on intervention adherence was not feasible due to the lack of quantifiable data, the role of adherence in improving prognosis cannot be overlooked. In the included studies, Dajpratham et al [48] reported that teleresistance exercise showed an adherence rate of 70%, higher than that of the control group [48]. A recently published meta-analysis also indicates that telerehabilitation is associated with higher levels of medical adherence compared to conventional care [74]. All these reflect the potential of DHIs in improving intervention adherence. Theoretically, the convenience, reminders, and engaging nature of DHIs are posited to enhance adherence. However, future research must prioritize the standardized measurement and reporting of adherence metrics (eg, login frequency and exercise completion rates) to quantitatively validate this claim and understand its relationship with clinical outcomes.

### Subgroup Analyses

Given the high heterogeneity, we performed subgroup analyses based on the type and duration of the intervention. Although only 1 study [46] used digital device-based DHIs (wearable devices), which prevented subgroup analysis by the type of intervention, the potential of digital device-based DHIs, especially wearable devices, to provide objective and continuous data on physical activity and movement patterns is immense [75,76]. It is reported that wearable devices have been used to collect body posture and movement data, providing real-time feedback to therapists to ensure rehabilitation programs are precisely tailored to individual needs [46]. It is evident that their role in enabling precise dosing of rehabilitation and detecting early signs of functional decline is a promising frontier [76]. The emerging field of wearable technology warrants dedicated investigation in the future, with more trials to isolate its specific contribution to the effectiveness of DHIs for hip fracture recovery [75].

Subgroup analyses stratified by intervention duration were performed to explore sources of heterogeneity in DHIs’ effects on hip function, with findings interpreted in light of heterogeneity and risk of bias. The test for subgroup differences revealed no statistically significant effects, indicating that intervention duration was not a contributor to the overall heterogeneity observed in the meta-analysis. The 3 months subgroup yielded a significant pooled treatment effect, which suggests that prolonged intervention duration may mitigate outcome variability by enabling cumulative rehabilitation gains and sustained behavior modification. Additionally, more uniform long-term intervention protocols across included trials may have reduced methodological heterogeneity. This result may also be related to the dose-response relationship of rehabilitation [77,78]. A longer intervention period allows for a greater cumulative “dose” of therapy, which is necessary to counteract the severe physical deconditioning post fracture.

Interestingly, the subgroup analysis on functional independence based on intervention duration yielded inconsistent findings, with interventions lasting <3 months appearing more effective than those lasting 3 months. Notably, however, each of the 3 individual studies [3,40,43] in the 3 months subgroup demonstrated a statistically significant improvement in functional independence for the DHIs group relative to controls. When pooled, these results became statistically insignificant—a discrepancy attributable to marked between-study heterogeneity within this subgroup. These 3 studies used the same measurement tools, and the degree of improvement evaluated by Prieto Moreno et al [47] was much higher than that of the other 2 studies [40,43], which might be related to their higher frequency, intervention, and usage rate. Prieto Moreno et al [47] had an intervention frequency of twice a week and reported a relatively high app usage rate. The other 2 studies, however, had no reports on usage rate or adherence rate and had a lower intervention frequency.

Collectively, these findings underscore the complexity of optimizing DHI protocols for older adults with hip fractures. While prolonged intervention duration conferred consistent benefits for hip function, its effects on functional independence appear contingent on ensuring sufficient intervention frequency and monitoring adherence. Future research should therefore prioritize standardized reporting of intervention dose and adherence metrics, alongside rigorous protocol design that specifies both duration and frequency of DHIs delivery, to clarify and replicate these subgroup-specific effects.

### Comparison with Prior Work

With the increasing popularity of DHIs, scholars are paying more and more attention to their effects in the field of bone and joint rehabilitation. Several similar systematic reviews and meta-analyses have been published. While Molina-Garcia et al [70] provided a broad overview of DHIs across musculoskeletal disorders (including hip fractures), our study focused specifically on older patients with hip fractures, which made the evidence we provided direct and specifically applicable to clinicians and rehabilitation experts managing older patients with hip fractures, enabling them to make more precise decisions regarding DHIs.

Two published systematic reviews and meta-analyses have focused on the older population with hip fractures, as we have. One of the studies was published by Pliannuom et al [21] in 2024, focusing only on the impact of DHIs on functional outcomes, while we simultaneously evaluated functional and psychological outcomes and intervention adherence [21]. Furthermore, their paper was limited by the number of studies (only 6 RCTs [39-44] were included), and they were unable to conduct subgroup analyses to further clarify which subgroups could benefit the most from DHIs. Compared with another meta-analysis by Xiao et al [74], our population mainly focuses on older patients with hip fractures rather than all patients after hip fractures and replacement surgeries. Furthermore, compared with Xiao et al [74], our analysis included more of the latest published studies and conducted a GRADE assessment on the included studies to ensure the quality of the studies and the reliability of the results of the meta-analysis [47,48]. Our study and the study by Xiao et al [74] both performed subgroup analyses of hip function based on intervention duration, and both found that a longer intervention period was associated with a greater improvement effect on hip function [74]. However, in the study by Xiao et al [74], the 6-month subgroups showed extremely high heterogeneity (*I*²=95%), indicating instability. In our study, the heterogeneity of the ≥3 months subgroup was low (*I*²=7%), which indicates that our findings are more robust and reliable.

However, our review indicates that only low-to-moderate certainty evidence supports the effects of DHIs on our outcomes of interest. This suggests that while there may be some positive impact, the evidence is not strong enough to draw definitive conclusions. The variability in study quality, sample sizes, and methodologies may contribute to the uncertainty, highlighting the need for more robust and well-conducted trials to strengthen the evidence base. Therefore, the results should be interpreted carefully, as future research might yield divergent findings.

### Strengths and Limitations

This meta-analysis followed the guidelines for performing rigorous systematic reviews. We also proposed a rigorous screening and search strategy to identify the most comprehensive literature. Sensitivity analyses revealed robust findings. Consequently, the results of our review support a widespread belief. However, there are also limitations in this systematic review. First, high statistical heterogeneity was observed in some results, which could not be fully explained by the subgroup analysis we prespecified. This may stem from the diversity of intervention components (such as types, dose, and intervention providers), control groups, and patient characteristics. Second, the limited number of studies on certain outcomes has restricted the statistical power and accuracy of estimates. Third, some studies had high risk or some concerns, particularly in terms of randomization and selection of reported results. This may have influenced the overall findings. Fourth, only one study [48] included in our review reported intervention adherence, which limits the quantitative evaluation for adherence of DHIs. Finally, as most of the research (61.5%) was conducted in China, the universality of the research results may be limited, and their applicability to other health care systems and cultures needs to be verified.

### Further Research and Clinical Implications

For further research, the sources of heterogeneity should be identified based on patient characteristics (eg, prefracture functional level), intervention dose (eg, frequency and duration), as our meta-analysis observed significant heterogeneity. The high heterogeneity observed across studies also underscores the need for standardized protocols of DHIs. Future research should also aim to identify the most effective components of DHIs and develop standardized intervention frameworks to reduce variability and improve the generalizability of findings. Second, as the existing RCTs lack assessment of psychological outcomes and intervention adherence, future RCTs should consider including these outcomes, which are particularly important for a more comprehensive evaluation of the effect of DHIs. Third, due to the limited number of studies using digital devices for DHIs, subgroup analysis based on intervention types was not achieved in this systematic review. Future research needs to explore the effects of digital devices to further clarify the impact of intervention types on improving the functional and psychological outcomes of patients with hip fractures. Fourth, future research should further explore the dose-response of exercise rehabilitation on the recovery of physical function after hip fractures to achieve the best intervention effect. Clinically, our research results indicate that DHIs have potential value in improving hip function and functional independence in older patients with hip fractures. Clinicians should consider incorporating DHIs into clinical practice and using them as routine care after hip fractures in older adults. This is of great significance for optimizing the allocation of medical resources and expanding the scope of rehabilitation services.

### Conclusions

This review incorporates the latest RCTs and comprehensively assesses both functional and psychological outcomes of DHIs in older patients with hip fractures, distinct from prior studies focusing solely on functional outcomes. While 95% CI supports the potential of DHIs to improve hip function and functional independence, the wide 95% PI indicating substantial real-world response variability calls for cautious interpretation, informing the design of targeted DHI-based rehabilitation regimens, and warrants further research into optimal techniques and dosages in clinical practice.

## Appendix

Multimedia Appendix 1 PRISIMA 2020 checklist.

Multimedia Appendix 2 PRISMA-S checklist.

Multimedia Appendix 3 Search strategy.

Multimedia Appendix 4 Sensitivity analysis.

## Abbreviations

DHI: digital health intervention
FBG: Functional Balance for Geriatric Patients
FIM: Functional Independence Measure
GRADE: Grading of Recommendations, Assessment, Development and Evaluation
HHS: Harris Hip Score
HKSJ: Hartung-Knapp-Sidik-Jonkman
HOOS: Hip Disability and Osteoarthritis Outcome Score
MFAC: Modified Functional Ambulatory Category
mHealth: mobile health
MOS: Medical Outcomes Study
PI: prediction interval
PRISMA: Preferred Reporting Items for Systematic Reviews and Meta-Analyses
PRISMA-S: Preferred Reporting Items for Systematic Reviews and Meta-Analyses literature search extension
QoL: quality of life
RCT: randomized controlled trial
SF-12: Short Form-12 Health Survey
SF-36: MOS 36-Item Short Form Health Survey
SMD: standardized mean difference
SPPB: Short Physical Performance Battery
TUG: Timed Up and Go Test
WHO: World Health Organization

## Data Availability

The datasets generated or analyzed during this systematic review are available from the corresponding author on reasonable request.
