# Supplementary material for: Effects of Digital Health Interventions on Functional and Psychological Outcomes in Older Patients With Hip Fractures: Systematic Review and Meta-Analysis of Randomized Controlled Trials
Source: J Med Internet Res. 2026 Mar 12;28:e79563. doi: 10.2196/79563 (PMC13022544; doi:10.2196/79563)
Supplement: Multimedia Appendix 3 [file jmir_v28i1e79563_app3.docx]

**Multimedia Appendix 3 Search strategy**

**Search date: 13^th^, November, 2025**

**Pubmed**

| Population | |
| --- | --- |
| #1 | "Hip Fractures"[MeSH Terms] |
| #2 | "hip fracture*"[Title/Abstract] OR "broken hip"[Title/Abstract] OR "intertrochanteric fracture*"[Title/Abstract] OR "subtrochanteric fracture*"[Title/Abstract] OR "trochanteric fracture*"[Title/Abstract] OR "pertrochanteric fracture*"[Title/Abstract] OR "trochanteric fracture*"[Title/Abstract] OR "femoral neck fracture*"[Title/Abstract] OR "femur neck fracture*"[Title/Abstract] OR "proximal femoral fracture*"[Title/Abstract] OR "proximal femur fracture*"[Title/Abstract] |
| #3 | #1 OR #2 |
| #4 | "Digital Health"[MeSH Terms] OR "Telemedicine"[MeSH Terms] OR "Telecommunications"[MeSH Terms] OR "Telerehabilitation"[MeSH Terms] OR "Internet"[MeSH Terms] OR "Computers"[MeSH Terms] OR "Virtual reality"[MeSH Terms] OR "Online Systems"[MeSH Terms] OR "Software"[MeSH Terms] OR "Electronic Mail"[MeSH Terms] OR "Electronic Health Records"[MeSH Terms] OR "Wearable Electronic Devices"[MeSH Terms] |
| #5 | "digital health"[Title/Abstract] OR "tele*"[Title/Abstract] OR "mhealth"[Title/Abstract] OR "m-health"[Title/Abstract] OR "ehealth"[Title/Abstract] OR "e-health"[Title/Abstract] OR "phone"[Title/Abstract] OR "smartphone"[Title/Abstract] OR "messag*"[Title/Abstract] OR "texting*"[Title/Abstract] OR "Short Message Service"[Title/Abstract] OR "SMS"[Title/Abstract] OR "facebook"[Title/Abstract] OR "twitter"[Title/Abstract] OR "application*"[Title/Abstract] OR "App"[Title/Abstract] OR "Apps"[Title/Abstract] OR "electronic mail*"[Title/Abstract] OR "e mail*"[Title/Abstract] OR "email*"[Title/Abstract] OR "sensor*"[Title/Abstract] OR "wearable*"[Title/Abstract] OR "Online"[Title/Abstract] OR "wireless"[Title/Abstract] OR "software"[Title/Abstract] OR "web"[Title/Abstract] OR "website*"[Title/Abstract] OR "network"[Title/Abstract] OR "digital"[Title/Abstract] OR "video"[Title/Abstract] OR "remote"[Title/Abstract] OR "virtural"[Title/Abstract] OR "mobile"[Title/Abstract] |
| #6 | #4 OR #5 |
| #7 | #3 AND #6 |
| Result | 3725 |

**Embase**

| Population | |
| --- | --- |
| #1 | 'hip fracture'/exp OR 'hip fracture*':ab,ti |
| #2 | 'hip fracture*':ab,ti OR 'hip broken':ab,ti OR 'intertrochanteric fracture*':ab,ti OR 'subtrochanteric fracture*':ab,ti OR 'pertrochanteric fracture*':ab,ti OR 'trochanteric fracture*':ab,ti OR 'femur trochlear fracture*':ab,ti OR 'femoral trochlear fracture*':ab,ti OR 'femoral neck fracture*':ab,ti OR 'femur neck fracture*':ab,ti OR 'proximal femoral fracture*':ab,ti OR 'proximal femur fracture*':ab,ti |
| #3 | #1 OR #2 |
| #4 | 'digital health'/exp OR 'telemedicine'/exp OR 'telehealth'/exp OR 'telecommunication'/exp OR 'telerehabilitation'/exp OR 'internet'/exp OR 'computer'/exp OR 'virtual reality'/exp OR 'online system'/exp OR 'mobile phone'/exp OR 'text messaging'/exp OR 'software'/exp OR 'mobile application'/exp OR 'e-mail'/exp OR 'electronic health record'/exp OR 'wearable device'/exp |
| #5 | 'digital health':ab,ti OR 'tele*':ab,ti OR 'mhealth':ab,ti OR 'm-health':ab,ti OR 'ehealth':ab,ti OR 'e-health':ab,ti OR 'phone':ab,ti OR 'smartphone':ab,ti OR 'messag*':ab,ti OR 'texting*':ab,ti OR 'short message service':ab,ti OR 'sms':ab,ti OR facebook:ab,ti OR twitter:ab,ti OR 'application*':ab,ti OR 'app':ab,ti OR 'apps':ab,ti OR 'electronic mail*':ab,ti OR 'e-mail*':ab,ti OR 'email*':ab,ti OR 'sensor*':ab,ti OR 'wearable*':ab,ti OR 'online':ab,ti OR 'wireless':ab,ti OR 'software':ab,ti OR 'web':ab,ti OR 'website*':ab,ti OR 'network':ab,ti OR 'digital':ab,ti OR 'video':ab,ti OR 'remote':ab,ti OR 'virtural':ab,ti OR 'mobile':ab,ti |
| #6 | #4 OR #5 |
| #7 | #3 AND #6 |
| Results | 8507 |

**WOS**

| Population | |
| --- | --- |
| #1 | TS=(“Hip Fracture*” or “Hip broken” or “Intertrochanteric Fracture*” or “Subtrochanteric Fracture*” or “Trochanteric Fracture*” or “Pertrochanteric Fracture*” or “Trochanteric Fracture*” or “Femur Trochlear Fracture*” or “Femoral Trochlear Fracture*” or “Femoral Neck Fracture*” or “Femur Neck Fracture*” or “Proximal Femoral Fracture*” or “Proximal Femur Fracture*”) |
| #2 | TS=(“digital health” OR “tele*” OR “mhealth” OR “m-health” OR “ehealth” OR “e-health” OR “phone” OR “smartphone” OR “messag*” OR “Texting*” OR “Short Message Service” OR “SMS” or facebook or twitter OR “Application*” OR “App” OR “Apps” OR “Electronic Mail*” OR “E-mail*” OR “Email*” OR “sensor*” OR “wearable*” OR “Online” OR “wireless” OR “software” OR “web” or “website*” OR “network” OR “digital” OR “video” OR “remote” OR “virtual” OR “mobile”) |
| #4 | #1 AND #2 AND #3 |
| Results | 3997 |

**Cochrane**

| Population | |
| --- | --- |
| #1 | MeSH descriptor: [Hip Fractures] explode all trees |
| #2 | (“Hip Fracture” or “Hip broken” or “Intertrochanteric Fracture” or “Subtrochanteric Fracture” or “Trochanteric Fracture” or “Pertrochanteric Fracture” or “Trochanteric Fracture” or “Femur Trochlear Fracture” or “Femoral Trochlear Fracture” or “Femoral Neck Fracture” or “Femur Neck Fracture” or “Proximal Femoral Fracture” or “Proximal Femur Fracture”):ti,ab,kw (Word variations have been searched) |
| #3 | #1 or #2 |
| #4 | MeSH descriptor: [Digital Health] explode all trees |
| #5 | MeSH descriptor: [Telecommunications] explode all trees |
| #6 | MeSH descriptor: [Telemedicine] explode all trees |
| #7 | MeSH descriptor: [Internet] explode all trees |
| #8 | MeSH descriptor: [Computers] explode all trees |
| #9 | MeSH descriptor: [Virtual Reality] explode all trees |
| #10 | MeSH descriptor: [Online Systems] explode all trees |
| #11 | MeSH descriptor: [Software] explode all trees |
| #12 | MeSH descriptor: [Medical Records] explode all trees |
| #13 | #4 OR #5 OR #6 OR #7 OR #8 OR #9 OR #10 OR #11 OR #12 |
| #14 | (“digital health” or "Telemedicine" OR “Virtual Medicine” OR “Mobile Health” OR “mHealth” OR “m-health” OR “Telehealth” OR “eHealth” OR “e-health” OR “Telecare” OR “Tele-Care” OR “Tele Care” OR "Telecommunication" or “Teleconference” OR “Phone” OR “telephone” OR “cell phone” or “smartphone” OR “mobile phone” OR “mobile telephone” OR “text messaging” OR “Texting” OR “Short Message Service” OR “SMS” OR “message” or facebook or twitters or "Telerehabilitation" OR “Virtual Rehabilitation” OR “Remote Rehabilitation” or "Mobile Application" OR “Application” OR “App” OR “Apps” OR “mobile device” OR “Electronic Mail” OR “E-mail” OR “Email” OR “sensor” OR “wearable” OR “Online” OR “wireless” OR “software” or “web” or “website” or “network” or “digital” or “video” or “remote”):ti,ab,kw (Word variations have been searched) |
| #15 | #13 OR #14 |
| #16 | #3 AND #15 |
| #17 | Fliter:trials |
| Results | 679 |

**APA PsycInfo**

| Population | |
| --- | --- |
| S1 | “Hip Fracture*” or “Hip broken” or “Intertrochanteric Fracture*” or “Subtrochanteric Fracture*” or “Trochanteric Fracture*” or “Pertrochanteric Fracture*” or “Trochanteric Fracture*” or “Femur Trochlear Fracture*” or “Femoral Trochlear Fracture*” or “Femoral Neck Fracture*” or “Femur Neck Fracture*” or “Proximal Femoral Fracture*” or “Proximal Femur Fracture*” |
| S2 | “digital health” OR “tele*” OR “mhealth” OR “m-health” OR “ehealth” OR “e-health” OR “phone” OR “smartphone” OR “messag*” OR “Texting*” OR “Short Message Service” OR “SMS” or facebook or twitter OR “Application*” OR “App” OR “Apps” OR “Electronic Mail*” OR “E-mail*” OR “Email*” OR “sensor*” OR “wearable*” OR “Online” OR “wireless” OR “software” OR “web” or “website*” OR “network” OR “digital” OR “video” OR “remote” OR “virtural” OR “mobile” |
| S3 | S1 AND S2 |
| Results | 173 |

**PEDro**

| Abstract & Title | fracture*+ |
| --- | --- |
| Body Part | thigh or hip+ |
| Method | clinical trial+ |
| Results 282 | |

**SinoMed**

| #1 | ((((("数字技术"[不加权:扩展]) OR "远程医学"[不加权:扩展] OR "远程康复"[不加权:扩展]) OR "虚拟现实"[不加权:扩展]) OR "远程护理"[不加权:扩展]) OR (( "远程康复"[常用字段:智能] OR "虚拟现实"[常用字段:智能] OR "远程医疗"[常用字段:智能] OR "应用程序"[常用字段:智能] OR "移动应用"[常用字段:智能] OR "移动医疗"[常用字段:智能]))) AND (("骨折"[常用字段:智能]) OR ("髋部"[常用字段:智能] OR "髋关节"[常用字段:智能] OR "股骨颈"[常用字段:智能] OR "转子间"[常用字段:智能] OR "粗隆间"[常用字段:智能]) OR ("髋部骨折"[常用字段:智能]) OR ("髋骨折"[不加权:扩展])) |  |
| --- | --- | --- |
| Results | 203 |  |

**CNKI**

| Strategy |
| --- |
| （主题：虚拟现实 + 移动医疗 + 远程 + 数字健康 + 数字医疗）AND（主题：髋部 + 髋关节 + 股骨颈 + 转子间 + 粗隆间） AND （主题：骨折） |
| Results 34 |

**Wanfang**

| Strategy |
| --- |
| 全部:(虚拟现实 or 移动医疗 or 远程 or 数字健康 or 数字医疗) and 全部:(髋部骨折 or 髋关节骨折 or 股骨颈骨折) |
| Results 108 |
