# Supplementary material for: Effects of Digital Health Interventions on Functional and Psychological Outcomes in Older Patients With Hip Fractures: Systematic Review and Meta-Analysis of Randomized Controlled Trials
Source: J Med Internet Res. 2026 Mar 12;28:e79563. doi: 10.2196/79563 (PMC13022544; doi:10.2196/79563)

**Multimedia Appendix 4 Sensitivity analysis and Funnel plot for hip function**

Sensitivity analysis on hip function


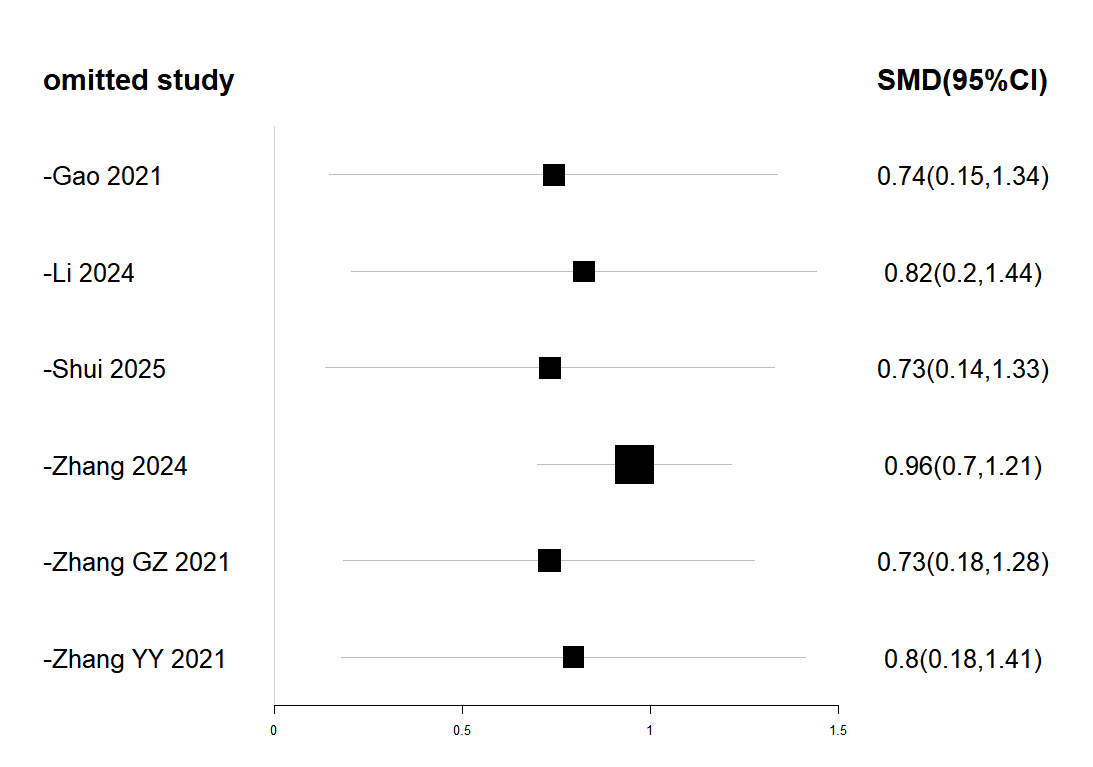


Sensitivity analysis on functional independence


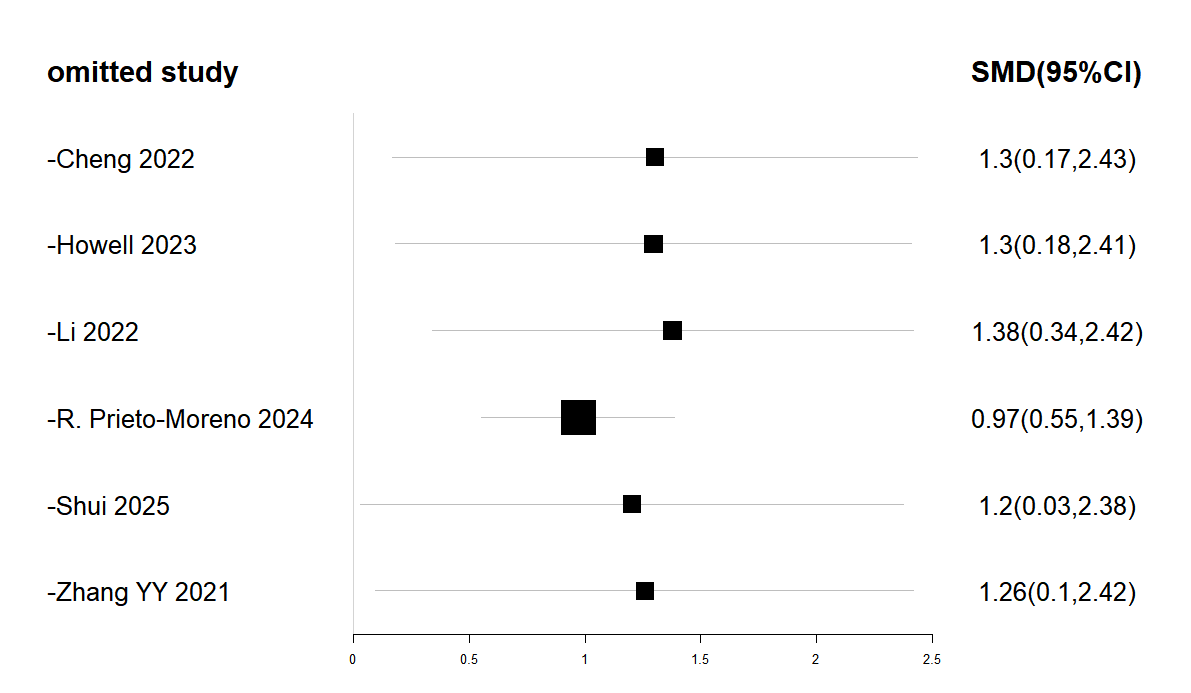


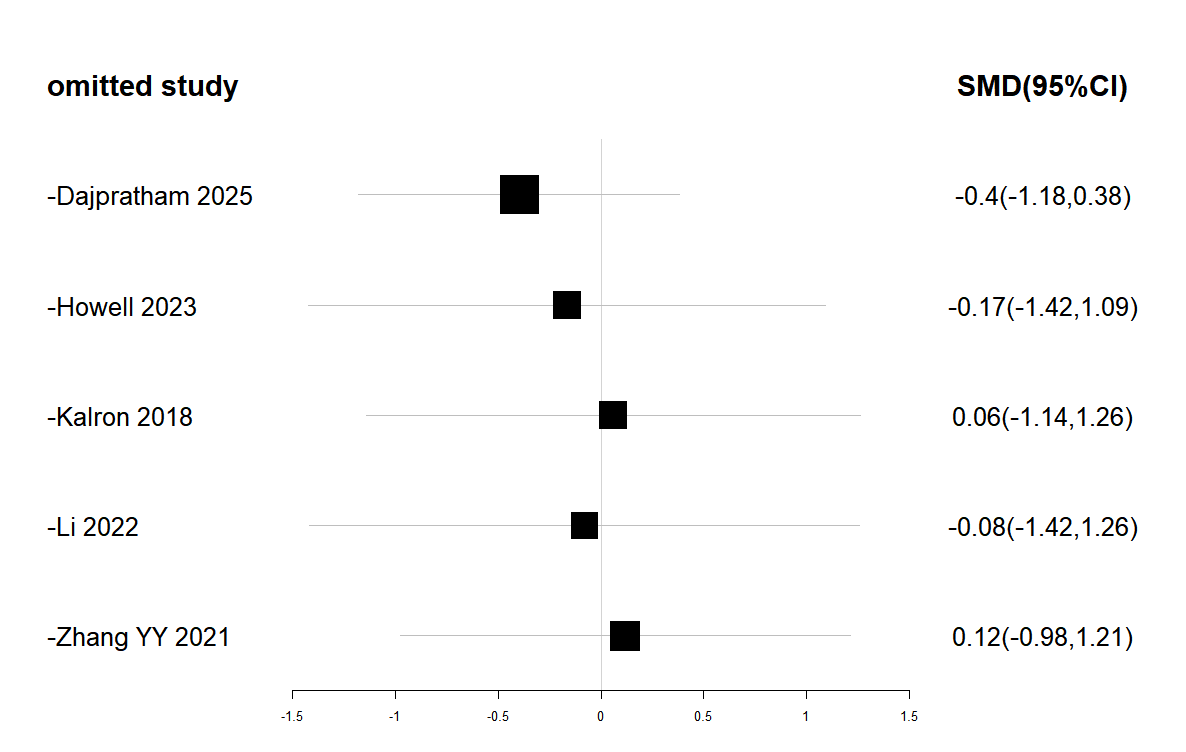
Sensitivity analysis on balance function and risk of falling


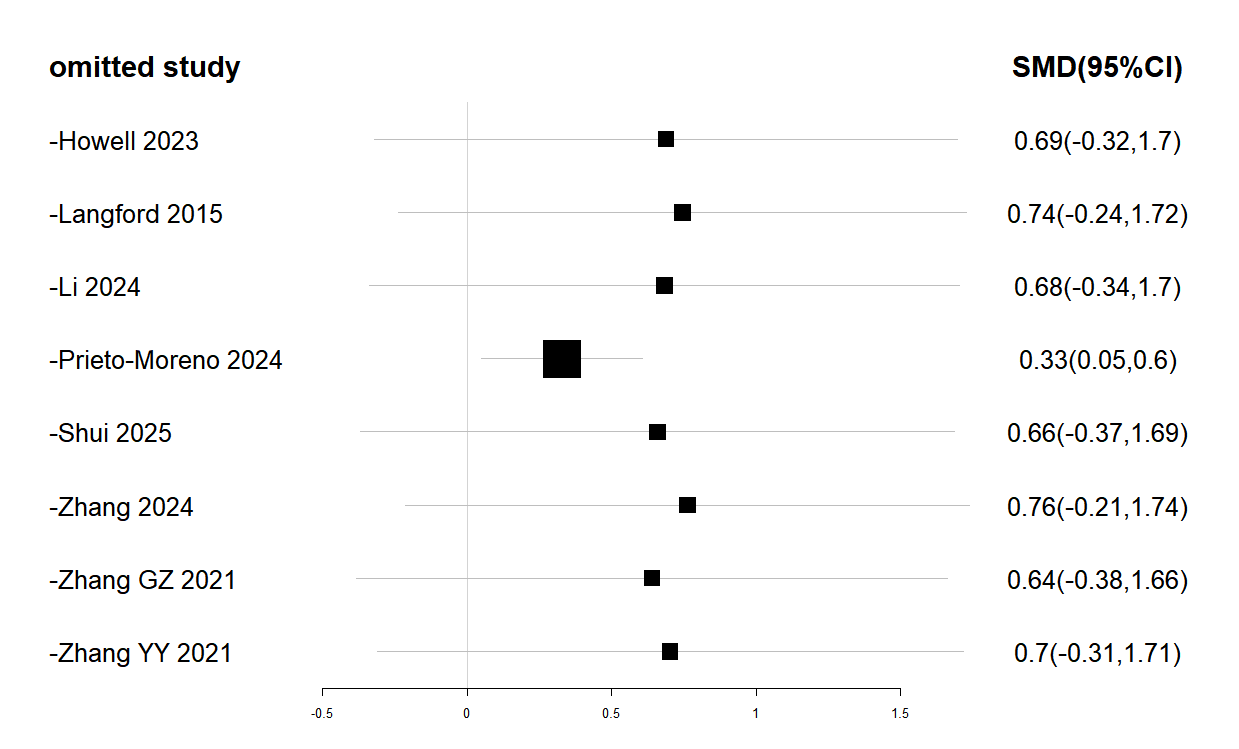
Sensitivity analysis on QoL

Funnel plot for hip function


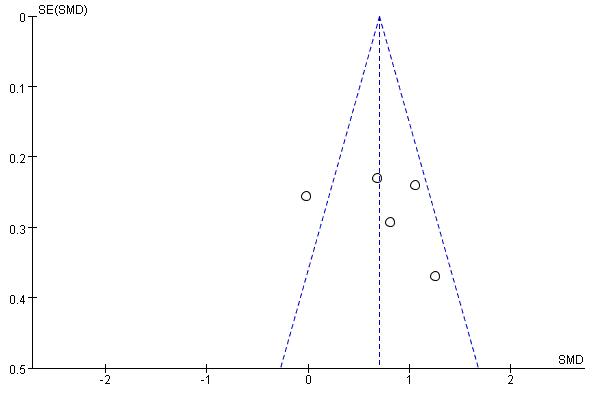

Supplement: Multimedia Appendix 4 [file jmir_v28i1e79563_app4.docx]
